# 2886. The Clinical Implications of High Echinocandin Persistence of a Major Fungal Pathogen and Development of a Rapid and Convenient Approach to Determine Echinocandin Persistence

**DOI:** 10.1093/ofid/ofad500.163

**Published:** 2023-11-27

**Authors:** Amir Arastehfar, Farnaz Daneshnia, Daniel Floyd, Michael Mansour

**Affiliations:** Massachusetts General Hospital, Boston, MA; Massachusetts General Hospital, Boston, MA; Massachusetts General Hospital, Boston, MA; Massachusetts General Hospital, Boston, MA

## Abstract

**Background:**

*Candida glabrata* is a major human fungal pathogen known for its rapid acquisition of antifungal resistance and its ability to replicate inside the macrophages. Echinocandins are the first-line antifungal drugs and the number of echinocandin resistant (ECR) *C. glabrata* isolates are on the rise. Echinocandin persistent (ECP) *C. glabrata* cells are one of the major drivers of ECR. Although bacterial isolates with high antibiotic persistence are associated with a higher rate of resistance and burden, it is not clear if ECP level in *C. glabrata* bears a clinical significance. Additionally, ECP level determination traditionally relies on labor-intensive and time-consuming approaches.

**Methods:**

Six *C. glabrata* isolates with similar minimum inhibitory concentrations of micafungin were included and their ECP was determined using traditional colony forming counting. An ex vivo primary macrophage model was used to assess the intracellular replication, the dynamic of killing, and the ECR colony rates of all isolates in the absence and presence of micafungin treatment. Two isolates, including a high and a low ECP *C. glabrata* isolates, were used to induce systemic infection and to evaluate the fungal burden and the rate of ECR colonies in spleen, liver, and kidney in the absence and presence of humanized dosage of micafungin (5mg/kg). A SYTOX-based assay was used as a proxy to determine the ECP levels of various *C. glabrata*.

**Results:**

We show that high ECP-Cg isolates better replicate inside the macrophages and establish systemic infection than low ECP isolate. Additionally, under echinocandin treatment high ECP-Cg isolates have a higher burden and exclusively develop ECR colonies in our ex vivo and systemic infection models. Finally, we report a convenient SYTOX-based assay that reliably and rapidly measures the ECP level.

**Conclusion:**

*C. glabrata* isolates with a higher ECP level are less effectively cleared by echinocandins and more likely to develop ECR and as such are clinically relevant. Our rapid and convenient SYTOX-based protocol reliably determines the ECP level and therefore has the potential to be used in clinical settings, high throughput screening studies, and has the potential to be extended to measure persistence level of various fungal pathogens.

**Disclosures:**

**Michael Mansour, MD, PhD**, Thermofisher: Grant/Research Support

